# Prognostic Value of Self-Reported Subjective Exercise Capacity in Patients With Acute Dyspnea

**DOI:** 10.1016/j.jacadv.2023.100342

**Published:** 2023-05-26

**Authors:** Maria Belkin, Desiree Wussler, Eleni Michou, Ivo Strebel, Nikola Kozhuharov, Zaid Sabti, Albina Nowak, Samyut Shrestha, Pedro Lopez-Ayala, Alexandra Prepoudis, Sabrina Stefanelli, Ibrahim Schäfer, Constantin Mork, Miriam Albus, Isabelle Danier, Cornelia Simmen, Tobias Zimmermann, Matthias Diebold, Tobias Breidthardt, Christian Mueller

**Affiliations:** a Cardiovascular Research Institute Basel (CRIB) and Department of Cardiology, University Hospital Basel, University of Basel, Basel, Switzerland; b Department of Internal Medicine, University Hospital Basel, Basel, Switzerland; c GREAT network, Rome, Italy; d Department of Cardiology, Liverpool Heart and Chest Hospital, Liverpool, United Kingdom; e Department of Endocrinology and Clinical Nutrition, University Hospital Zurich, Zurich, Switzerland; f Division of Internal Medicine, University Psychiatry Clinic Zurich, Zurich, Switzerland

**Keywords:** acute dyspnea, acute heart failure, Duke Activity Status Index, self-reported exercise capacity

## Abstract

**Background:**

Self-reported exercise capacity is a well-established prognostic measure in stable ambulatory patients with cardiac and pulmonary disease.

**Objectives:**

The authors aimed to directly compare the prognostic accuracy of quantified self-reported exercise capacity using the Duke Activity Status Index (DASI) with the established objective disease-severity marker B-type natriuretic peptide (BNP) in patients presenting with acute dyspnea to the emergency department.

**Methods:**

The DASI was obtained in a prospective multicenter diagnostic study recruiting unselected patients presenting with acute dyspnea to the emergency department. The prognostic accuracy of DASI and BNP for 90-day and 720-day all-cause mortality was evaluated using C-index.

**Results:**

Among 1,019 patients eligible for this analysis, 75 (7%) and 297 (29%) patients died within 90 and 720 days after presentation, respectively. Unadjusted hazard ratios (HRs) and multivariable adjusted hazard ratios (aHRs) for 90- and 720-day mortality increased continuously from the fourth (best self-reported exercise capacity) to the first DASI quartile (worst self-reported exercise capacity). For 720-day mortality the HR of the first quartile vs the fourth was 9.1 (95% CI, 5.5-14.9) vs (aHR: 6.1, 95% CI: 3.7-10.1), of the second quartile 6.4 (95% CI: 3.9-10.6) vs (aHR: 4.4, 95% CI: 2.6-7.3), while of the third quartile the HR was 3.2 (95% CI: 1.9-5.5) vs (aHR: 2.4, 95% CI: 1.4-4.0). The prognostic accuracy of the DASI score was high, and higher than that of BNP concentrations (720-day mortality C-index: 0.67 vs 0.62; *P* = 0.024).

**Conclusions:**

Quantification of self-reported subjective exercise capacity using the DASI provides high prognostic accuracy and may aid physicians in risk stratification. (Basics in Acute Shortness of Breath EvaLuation [BASEL V] Study [BASEL V]; NCT01831115)

Objectively quantified exercise capacity is a well-established prognostic factor in stable ambulatory patients with cardiovascular and/or pulmonary diseases.^1^, ^2^, ^3^ For example, peak oxygen uptake measured by cardiopulmonary exercise testing and the 6-minute walk test aid physicians in the risk stratification of ambulatory patients with cardiac or pulmonary disease.^3^, ^4^, ^5^ Quantitative assessment of self-reported subjective exercise capacity has been evaluated as a simple alternative tool to potentially even better reflect the impact of exercise capacity on health-related quality of life.^6^, ^7^, ^8^ Likely the best validated one is the Duke Activity Status Index (DASI), a simple self-assessment tool with 12 questions for estimating exercise capacity.^9^ DASI-scores, with higher values indicating higher exercise capacity, correlate very well with peak oxygen uptake and are validated measures of functional status, particularly in outpatients with heart failure and in the preoperative setting.^9^^,^^10^

It is unknown, whether beyond providing important insights regarding functional health-related quality of life, quantitative assessment of self-reported subjective exercise capacity using the DASI may also help physicians in the risk stratification of patients presenting to the emergency department (ED) with acute dyspnea due to either acute heart failure (AHF) or pulmonary disease. Given the lack of established disease-independent tools for accurately risk stratifying patients with acute dyspnea, the DASI may well have clinical utility. Therefore, we aimed to test this hypothesis by directly comparing the prognostic accuracy of quantified self-reported exercise capacity using the DASI with the established objective disease-severity marker B-type natriuretic peptide (BNP) in a large prospective multicenter study.

## Methods

### Study design and population

BASEL V (Basics in Acute Shortness of Breath EvaLuation) was a prospective, multicenter, diagnostic, and prognostic study enrolling adult patients presenting with acute dyspnea to the ED in 2 University Hospitals in Switzerland (Basel & Zurich).^11^, ^12^, ^13^, ^14^, ^15^ Patients were included irrespective of renal function, whereas patients with terminal kidney failure on chronic dialysis were excluded. For this analysis, patients were eligible if they had completed the DASI questionnaire within the first days after presentation.

The investigation conforms with the principles outlined in the Declaration of Helsinki and the study was approved by the local ethics committee. The authors designed the study, gathered, and analyzed the data according to the STARD (Standards for Reporting Diagnostic accuracy studies) guidelines for studies of diagnostic accuracy.

### Quantified self-reported exercise capacity: DASI

**Figure 1:**
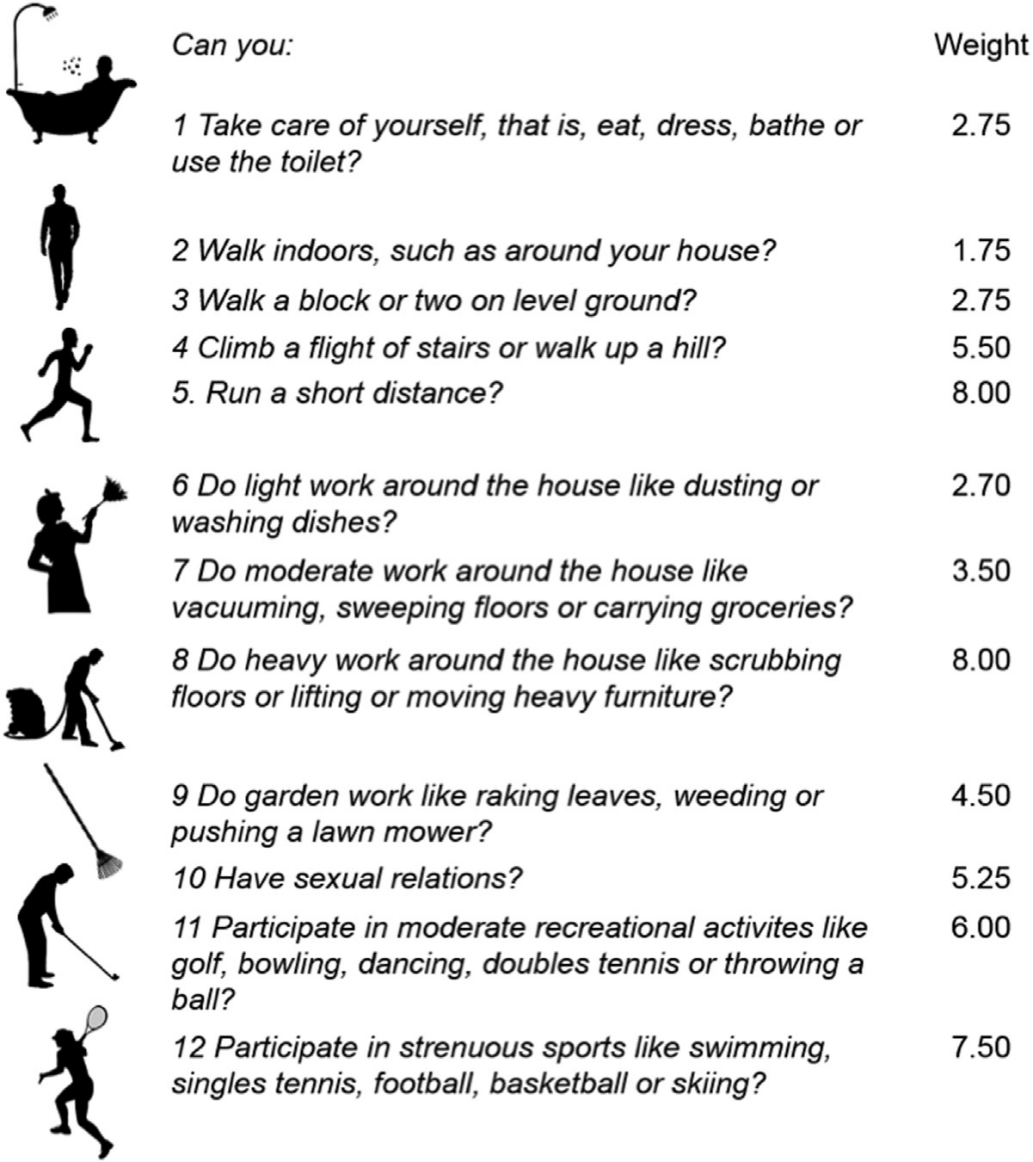
**Original Duke Activity Status Index** 12 original DASI questions and corresponding points which result in the DASI-score ranging from 0 (lowest) to 58.2 (highest exercise capacity). DASI = Duke Activity Status Index.

After patients had provided written informed consent, they received paper forms with 12 questions regarding exercise capacity during daily life representing the DASI and were asked to complete the forms within the first days of hospitalization. Initially, the original 12-item DASI version was translated into German and used (Figure 1).^9^ After 2 patients were severely offended by the question regarding sexual activities, in consultation with the ethics committee we decided to omit this question from all further questionnaires. The maximum score of this modified version of the DASI, which was later applied for all patients in this analysis, was 52.95 vs 58.2 in the original version.

### Adjudication of final diagnosis

The final diagnosis of the main disorder responsible for acute dyspnea was centrally adjudicated by 2 independent cardiologists/internists who had access to all patients' medical records including clinical history, physical examination, 12-lead electrocardiogram, laboratory findings, chest x-ray, echocardiography, lung function testing, computed tomography, the response to therapy, and also autopsy data for patients who had died in hospital. All laboratory findings obtained through the clinician’s routine diagnostic workup were available for this study. These findings included one of the natriuretic peptides (BNP or N-Terminal pro-BNP [NT-proBNP]) that current guidelines recommended for diagnosing AHF with a Class I recommendation.^1^^,^^2^ In situations of disagreement about the final diagnosis, cases were reviewed and adjudicated by a third cardiologist.

### Follow-up

Patients were contacted 3, 12, and 24 months after discharge by telephone or in written form. Specific questions regarding dyspnea, possible rehospitalizations, and relevant diagnostics (eg, electrocardiogram, echocardiography …) performed after discharge were assessed. Furthermore, patients were asked to fill out another DASI questionnaire which was sent in paper form. Information regarding death during follow-up was obtained from the hospital medical records, the general practitioner, and the national mortality registry.

### General statistical methods

Continuous variables are presented as median (IQR) and categorical variables are expressed by numbers (percentages). Linear regression and Cochran–Armitage tests were used to calculate *P* values for trend across the quartiles for continuous and categorical characteristics, respectively.^16^^,^^17^ All hypothesis testing was 2-tailed and *P* values of <0.05 were considered to indicate statistical significance. Adjustment for multiple testing was not performed. Statistical analysis was operated using SPSS for Windows 26.0 (SPSS Inc) and R statistical Software Version 3.4.3 (MathSoft).

### DASI-score for risk stratification

Unadjusted and multivariable adjusted Cox regression analyses were performed to quantify the independent effect of the DASI on 90- and 720-day all-cause mortality. The DASI was considered as a continuous score as well as stratified into quartiles with the fourth DASI-quartile including patients with the highest exercise capacity and serving as reference group. When treated as a continuous variable DASI score was reversed, thus a higher score indicated lower exercise capacity. The Cox regression model of 90-day mortality was adjusted for previously published covariates, further referred to as the “compact model”, including age, the natural logarithm blood urea nitrogen, hemoglobin level, the natural logarithm of NT-proBNP concentration at presentation, beta-blocker intake on entry, and besides, sex was added.^18^ Higher event rate allowed to additionally adjust the Cox regression model of 720-day mortality, based on relevant clinical findings and medical knowledge, to: systolic blood pressure and peripheral oxygen saturation at presentation, leg edema on entry, history of hypertension, diabetes mellitus, coronary artery disease, atrial fibrillation, previous heart failure and obstructive pulmonary disease, serum creatinine and sodium level, intake of angiotensin converting enzyme inhibitors/angiotensin receptor blockers, and diuretics.^18^^,^^19^ Missing predictor values were imputed using the Markov Chain Monte Carlo method. The number of imputed datasets was 20. The Cox regression analysis was performed on all 20 data sets and the results were pooled using Rubin’s rules.^20^ Findings were confirmed in a sensitivity analysis using the original data. Schoenfeld’s global test was used to test the proportional hazards assumption of the Cox regression models. Cox regression models were internally bootstrap validated. Kaplan–Meier curves of 90- and 720-day all-cause mortality were plotted, and comparisons were performed by log-rank tests. Censored patients were displayed in tables including number at risk with the use of “survminer” package R statistical software.

### Prognostic accuracy

Secondary analysis included the comparison of the prognostic accuracy between the continuous DASI score and BNP, an established objective disease-severity marker in patients presenting with acute dyspnea to the ED (primary analysis), as well as between the DASI score and NT-proBNP, which in addition to quantifying hemodynamic cardiac stress similar to BNP seems also to be associated with renal dysfunction as another prognostic variable.^13^^,^^21^, ^22^, ^23^, ^24^, ^25^ The prognostic accuracy of the DASI score was also compared to a previously described risk score derived from the compact mortality model, further referred to as the “Voors score”, ranging from 0 to 5.^18^ Calculations were performed in the original, not imputed data. Moreover, the internal bootstrap validation of the fully adjusted model (19 variables plus DASI score) was performed in a single imputed dataset. All prognostic accuracies were reported as optimism corrected C-indices. Correlated C-indices were compared using the “compareC” package R statistical software.^26^ Calibration curves showed observed vs predicted probability for 720-day all-cause mortality of BNP level on admission, the Voors score, the DASI quartiles, and the DASI score are part of the supplemental material (“rms”).^27^ Clinical usefulness was assessed by a decision curve analysis (“rmda”) calculating the clinical net benefit (true-positive counts minus false-positive counts weighted by the respective threshold probability). For a specific threshold probability, a larger net benefit indicates a greater number of true-positive predictions without increase of false positives. Possible time dependencies were studied using time-dependent area under the receiver operating characteristic curve which accommodates censored data (“timeROC”).^28^

### Subgroup analyses

Finally, a subgroup analysis was performed in patients with an adjudicated final diagnosis of AHF. Interaction tests were conducted between gender and the DASI score to evaluate a potential sex-specific effect on mortality in general and in inpatients with adjudicated AHF in particular.

## Results

Among 2,153 patients enrolled in BASEL V, 1,019 patients with a median age of 74 years comprising 43% women were eligible for this analysis, as they completed the DASI questionnaire shortly after presentation (median, 2 days; IQR: 1-5 days) (Supplemental Figure 1). Overall, baseline patient characteristics were comparable among patients completing the DASI questionnaire vs those who did not (Supplemental Table 1). The median-modified DASI score was 24.95 (IQR: 15.45-39.45). The most common adjudicated final diagnosis as the cause of acute dyspnea was AHF in 529 (52%) patients. Table 1 displays the baseline characteristics of patients included in this analysis and Supplemental Table 2 shows an overview of the covariates with missing values and summary statistics.

**Table 1:** Baseline Characteristics in the Overall Cohort Grouped According to DASI Quartiles

	All Patients (N = 1,019)	First Quartile (n = 252)	Second Quartile (n = 248)	Third Quartile (n = 268)	Fourth Quartile (n = 251)	*P* Value for Trend
DASI-score	24.95 (15.45-39.45)	7.25 (4.5-12.7)	18.95 (15.45-18.95)	31.45 (26.95-34.95)	52.95 (45.45-52.95)	<0.001
Age, y	74 (61-82)	75 (65-82)	76 (65-83)	76 (63-82)	66 (52-77)	<0.001
Female	436 (43)	117 (46)	110 (44)	114 (43)	95 (38)	0.049
Body mass index, kg/m^2^	26 (22-30)	26 (22-31)	25 (22-30)	26 (22-30)	26 (23-30)	0.708
History						
Hypertension	689 (68)	196 (78)	177 (71)	186 (69)	130 (52)	<0.001
Diabetes	239 (24)	77 (31)	56 (23)	67 (25)	39 (16)	<0.001
Ever a smoker	684 (68)	178 (72)	168 (69)	173 (65)	165 (67)	0.135
Coronary artery disease	355 (35)	97 (39)	100 (40)	94 (35)	64 (26)	0.001
History of heart failure	353 (35)	108 (43)	105 (42)	95 (36)	45 (18)	<0.001
Atrial fibrillation	301 (30)	89 (36)	75 (30)	87 (33)	48 (19)	<0.001
COPD/asthma	357 (35)	116 (46)	86 (35)	85 (33)	70 (28)	<0.001
Vital signs and symptoms on admission						
Systolic blood pressure, mm Hg	138 (122-155)	134 (119-151)	137 (120-152)	139 (123-156)	139 (125-159)	0.010
Heart rate, beats/min	90 (76-107)	92 (75-108)	90 (77-103)	88 (75-106)	92 (76-110)	0.306
SpO_2_, %	96 (93-98)	95 (91-97)	96 (93-98)	96 (94-98)	96 (93-98)	0.013
Body temperature, °C	37.1 (36.7-37.6)	37.1 (36.7-37.6)	37.0 (36.6-37.6)	37.1 (36.6-37.7)	37.1 (36.7-37.7)	0.041
Leg edema	430 (42)	132 (53)	115 (47)	103 (39)	78 (32)	<0.001
First inhospital laboratory findings						
BUN, mmol/L	7.5 (5.2-11.1)	8.2 (5.6-12.7)	8.2 (5.7-12.5)	7.9 (5.2-11.3)	6.5 (4.8-9.0)	<0.001
Hemoglobin, g/L	132 (117-146)	127 (114-142)	127 (111-142)	133 (117-146)	139 (130-150)	<0.001
Creatinine, μmol/L	88 (69-119)	89 (69-130)	97 (73-135)	89 (67-120)	83 (69-100)	0.002
Sodium, mmol/L	139 (136-141)	138 (135-141)	139 (136-141)	139 (136-141)	139 (137-141)	0.024
Potassium, mmol/L	4.1 (3.8-4.4)	4.1 (3.7-4.6)	4.1 (3.8-4.5)	4.1 (3.8-4.4)	4.1 (3.8-4.3)	0.197
NT-proBNP, ng/L	1,229 (229-5,214)	2,027 (381-6,308)	1,757 (378-5,606)	1,315 (236-5,281)	404 (92-2,389)	<0.001
Medication on admission						
ACEI/ARBs	509 (51)	141 (57)	136 (56)	146 (56)	86 (35)	<0.001
Beta-blockers	435 (44)	120 (49)	118 (49)	119 (45)	78 (31)	<0.001
Diuretics	522 (52)	155 (63)	142 (59)	144 (55)	81 (33)	<0.001

Values are median (IQR) or n (%). Patients stratified according to DASI into quartiles. *P* values for trend calculated using Cochran–Armitage test for categorical and linear regression for continuous variables. *P* < 0.05 were considered statistically significant. All hypothesis testing was 2-tailed.

ACEI = angiotensin converting enzyme inhibitor; ARB = aldosterone receptor blocker; BUN = blood urea nitrogen; COPD = chronic obstructive pulmonary disease; DASI = Duke Activity Status Index; NT-proBNP = N-terminal pro-B-type natriuretic peptide; SpO_2_ = peripheral oxygen saturation.

### DASI score for risk stratification

**Figure 2:**
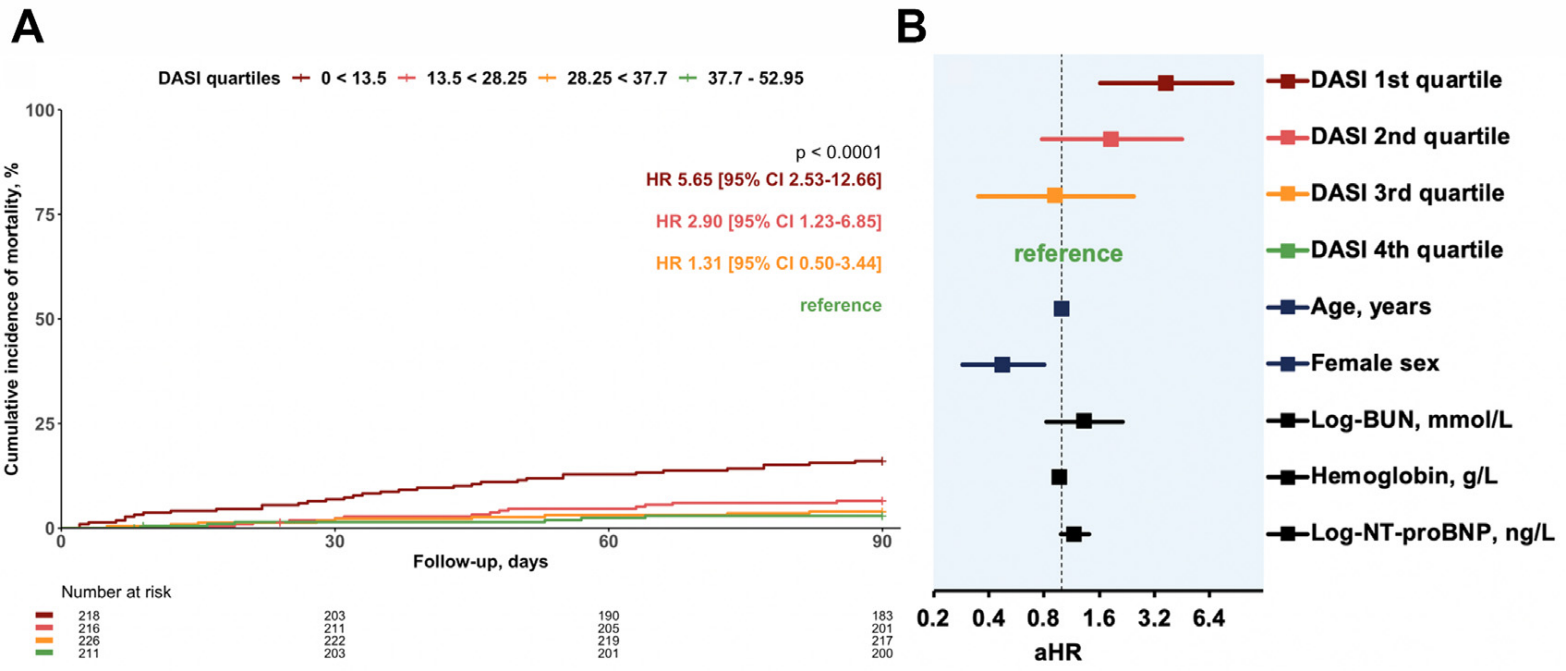
**DASI in Predicting Future Risk of 90-Day Death From Any Cause** Patients stratified according to DASI into quartiles and referenced to fourth quartile (highest self-reported exercise capacity). **(A)** Kaplan–Meier curves with *P* value (log-rank test), unadjusted HR with corresponding 95% CI and numbers at risk. **(B)** Adjusted HRs on logarithmic scale. Adjustments made for age, sex, natural log of blood urea nitrogen (mmol/L), hemoglobin (g/L), and natural log of N-terminal pro B-type natriuretic peptide (ng/L). Squares represent HRs; horizontal lines, 95% CI; vertical line, a HR of 1.00. aHR = adjusted hazard ratio; BUN = blood urea nitrogen; CI = confidence interval; DASI = Duke Activity Status Index; HR = hazard ratio; NT-proBNP = N-terminal pro-B-type natriuretic peptide.

**Figure 3:**
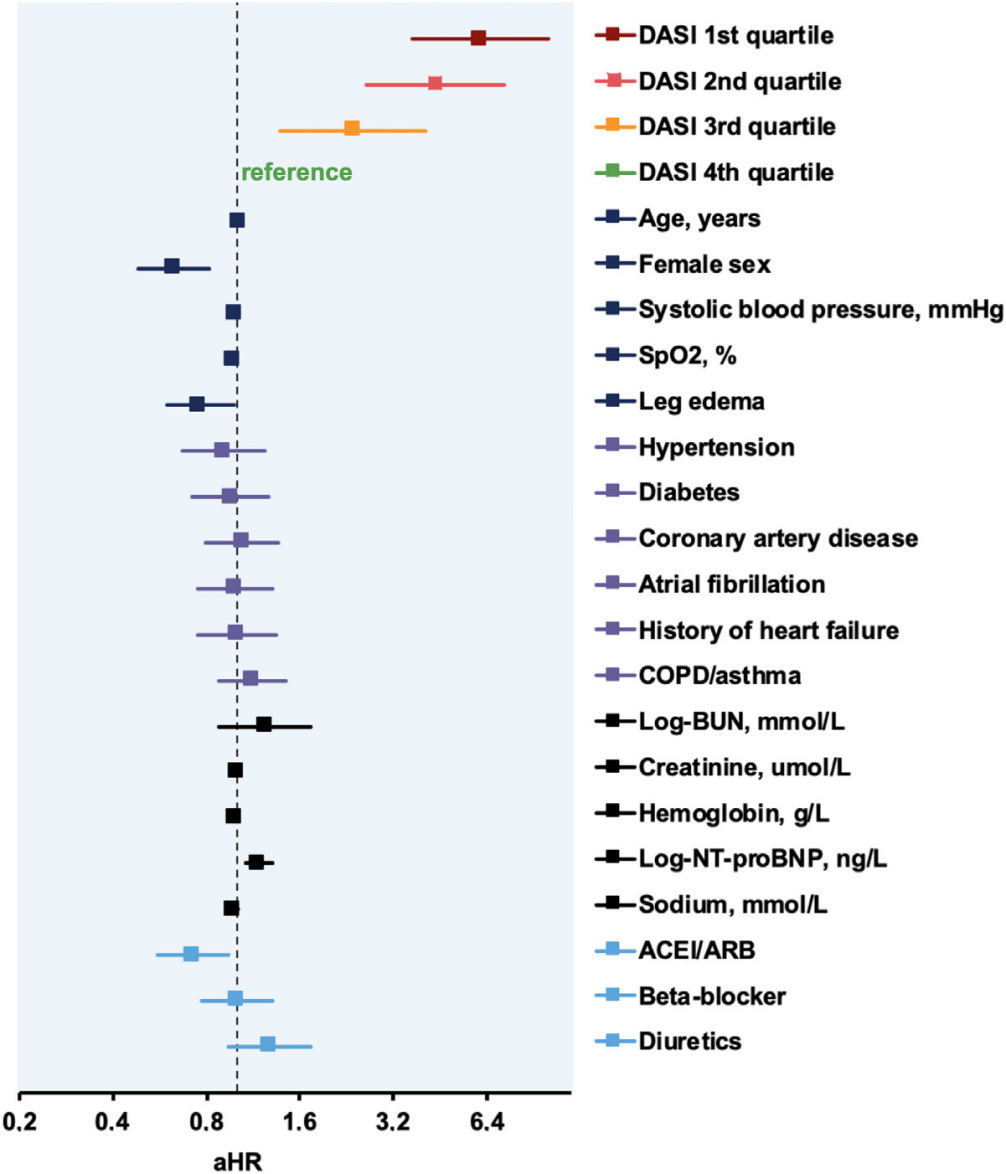
**DASI in Predicting Future Risk of 720-Day Death From Any Cause** Patients stratified into DASI quartiles and referenced to fourth quartile (highest self-reported exercise capacity). Adjusted hazard ratios (aHR) illustrated on logarithmic scale. Adjustments made for age (years), sex, systolic blood pressure (mm Hg), peripheral oxygen saturation (SpO_2_, %), leg edema on presentation, history of hypertension, diabetes, coronary artery disease, atrial fibrillation, heart failure, chronic obstructive pulmonary disease/asthma, natural logarithm of blood urea nitrogen (mmol/L), levels of serum creatinine (μmol/L), hemoglobin (g/L) and natural logarithm of N-terminal pro B-type natriuretic peptide (ng/L), sodium (mmol/L), intake of angiotensin-converting enzyme inhibitors/angiotensin receptor blockers (ACEI/ARB), beta-blockers, and diuretics on admission. **Squares** represent HRs; **horizontal lines**, 95% confidence intervals; **vertical line**, a HR of 1.00. BUN = blood urea nitrogen; COPD = chronic obstructive pulmonary disease; DASI = Duke Activity Status Index; NT-proBNP = N-terminal pro-B-type natriuretic peptide.

Within 90 and 720 days of follow-up, 75 (7%) and 297 (29%) patients died, respectively. The unadjusted hazard ratios (HRs) and the adjusted hazard ratios (aHR) for 90- and 720-day mortality increased continuously from the fourth quartile (best self-reported exercise capacity reference, DASI score: 40.45-52.95, n = 251) to the third quartile (DASI score: 24.95 < 40.45, n = 268), to the second quartile (DASI score 15.45 < 24.95, n = 248) up to the first quartile (worst self-reported exercise capacity, DASI score: 0-15.25, n = 252) (Figure 2, Central Illustration, Tables 2 and 3). For 720-day mortality the HR of the first vs the fourth quartile was 9.1 (95% CI: 5.5-14.9) and the aHR 6.1 (95% CI: 3.7-10.1), the HR of the second quartile was 6.4 (95% CI: 3.9-10.6) and the aHR: 4.4 (95% CI: 2.6-7.3), while the HR of the third was 3.2 (95% CI: 1.9-5.5) and the aHR 2.4 (95% CI: 1.4-4.0) (Figure 3). When analyzing DASI as a continuous variable again the HR and aHR indicated a higher risk for mortality for lower DASI scores, and therefore, lower exercise capacity (Supplemental Tables 3 and 4). For 90-day mortality the HR and aHR were 1.05 (95% CI: 1.03-1.07) and 1.04 (95% CI: 1.02-1.07), respectively. These findings were confirmed in sensitivity analyses only including not imputed data with 851 and 823 patients for the 90-day and 720-day analyses, respectively (data not shown).

**Central Illustration:**
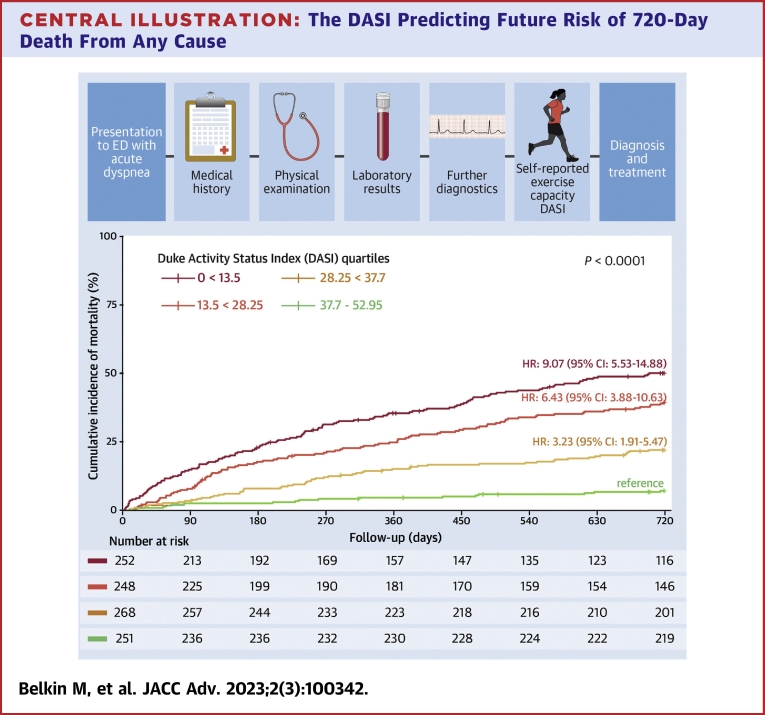
**The DASI Predicting Future Risk of 720-Day Death From Any Cause** Patients stratified into DASI quartiles and referenced to fourth quartile (highest self-reported exercise capacity). Kaplan–Meier curves with *P* value (log-rank test), unadjusted HRs with corresponding 95% CI and numbers at risk. DASI = Duke Activity Status Index; ED = emergency department.

**Table 2:** Duke Activity Status Index in Predicting Future Risk of 90-Day Death From Any Cause

	Univariable Analyses			Multivariable Analysis		
	HR	95% CI	*P* Value	HR	95% CI	*P* Value
Age (y)	1.034	1.015-1.054	0.001	1.016	0.994-1.038	0.158
Female	0.578	0.354-0.944	0.028	0.481	0.289-0.802	0.005
Log-BUN (mmol/L)	2.533	1.719-3.734	<0.001	1.337	0.829-2.155	0.233
Hemoglobin (g/L)	0.974	0.964-0.983	<0.001	0.982	0.972-0.993	0.001
Log-NT-proBNP (ng/L)	1.407	1.226-1.616	<0.001	1.182	0.992-1.408	0.062
Beta-blockers	1.129	0.717-1.776	0.601	0.680	0.426-1.086	0.106
DASI fourth quartile^a^	Reference					
DASI third quartile^b^	1.310	0.499-3.442	0.583	0.934	0.352-2.480	0.892
DASI second quartile^c^	2.897	1.225-6.852	0.015	1.874	0.779-4.511	0.161
DASI first quartile^d^	5.654	2.525-12.662	<0.001	3.717	1.629-8.482	0.002

Unadjusted and adjusted Cox regression analyses with patients (n = 1,019) stratified according to DASI into quartiles and referenced to fourth quartile. Adjustments made for age (y), sex, natural log-transformed BUN (mmol/L), hemoglobin level (g/L), natural log of NT-proBNP (ng/L) concentrations at presentation and intake of beta-blockers on admission.

BUN = blood urea nitrogen; CI = confidence interval; DASI = Duke Activity Status Index; HR = hazard ratio; NT-proBNP = N-terminal pro-B-type natriuretic peptide.

a High functional exercise capacity (fourth quartile): DASI 40.45 to 52.95 (n = 251).

b Moderately high functional exercise capacity (third quartile): DASI 24.95 < 40.45 (n = 268).

c Moderately low functional exercise capacity (second quartile): DASI 15.45 < 24.95 (n = 248).

d Low functional exercise capacity (first quartile): DASI 0 < 15.45 (n = 252).

**Table 3:** Duke Activity Status Index in Predicting Future Risk of 720-Day Death From Any Cause

	Univariable Analyses			Multivariable Analysis		
	HR	95% CI	*P* Value	HR	95% CI	*P* Value
Age (y)	1.046	1.035-1.057	<0.001	1.031	1.018-1.044	<0.001
Female	0.782	0.607-0.988	0.040	0.628	0.485-0.813	<0.001
Systolic blood pressure (mm Hg)	0.986	0.982-0.991	<0.001	0.992	0.987-0.997	0.002
SpO_2_ (%)	0.975	0.957-0.995	0.012	0.981	0.960-1.002	0.081
Leg edema	1.428	1.138-1.793	0.002	0.764	0.597-0.978	0.032
Hypertension	1.571	1.203-2.051	0.001	0.913	0.673-1.238	0.559
Diabetes	1.289	1.001-1.661	0.049	0.955	0.717-1.273	0.755
Coronary artery disease	1.748	1.391-2.196	<0.001	1.041	0.792-1.368	0.774
Atrial fibrillation	1.700	1.347-2.145	<0.001	0.986	0.751-1.295	0.920
History of heart failure	2.076	1.653-2.607	<0.001	1.006	0.751-1.347	0.969
COPD/asthma	1.169	0.926-1.476	0.189	1.130	0.882-1.449	0.333
Log-BUN (mmol/L)	2.073	1.704-2.522	<0.001	1.235	0.878-1.738	0.224
Creatinine (μmol/L)	1.003	1.002-1.005	<0.001	0.999	0.996-1.001	0.329
Hemoglobin (g/L)	0.981	0.976-0.986	<0.001	0.990	0.984-0.996	0.002
Log-NT-proBNP (ng/L)	1.353	1.266-1.447	<0.001	1.181	1.073-1.299	0.001
Sodium (mmol/L)	0.983	0.957-1.009	0.188	0.983	0.957-1.010	0.206
ACEI/ARBs	1.271	1.010-1.598	0.041	0.724	0.558-0.939	0.015
Beta-blockers	1.540	1.226-1.935	<0.001	1.002	0.772-1.302	0.986
Diuretics	2.365	1.845-3.031	<0.001	1.274	0.936-1.732	0.123
**DASI fourth quartile**^a^	**reference**					
**DASI third quartile**^b^	**3.230**	**1.906-5.476**	**<0.001**	**2.369**	**1.388-4.042**	**0.002**
**DASI second quartile**^c^	**6.425**	**3.883-10.632**	**<0.001**	**4.370**	**2.613-7.308**	**<0.001**
**DASI first quartile**^d^	**9.071**	**5.530-14.878**	**<0.001**	**6.081**	**3.656-10.115**	**<0.001**

Unadjusted and adjusted Cox regression analyses with patients (n = 1,019) stratified according to DASI into quartiles and referenced to fourth quartile. Adjustments made for age (y), sex, systolic blood pressure (mm Hg), and peripheral oxygen saturation (%) on presentation, leg edema, history of hypertension, diabetes mellitus, coronary artery disease, atrial fibrillation, heart failure and chronic obstructive lung disease/asthma, natural log of blood urea nitrogen (mmol/L), creatinine level (μmol/L), hemoglobin level (g/L), natural log of NT-proBNP (ng/L) concentrations and sodium level (mmol/L) at presentation and intake of ACEI/ARBs, beta-blockers, and diuretics at admission.

ACEI = angiotensin converting enzyme inhibitor; ARB = aldosterone receptor blocker; BUN = blood urea nitrogen; COPD = chronic obstructive pulmonary disease; DASI = Duke Activity Status Index; NT-proBNP = N-terminal pro-B-type natriuretic peptide; SpO_2_ = peripheral oxygen saturation.

a High functional exercise capacity (fourth quartile): DASI 40.45 to 52.95 (n = 251).

b Moderately high functional exercise capacity (third quartile): DASI 24.95 < 40.45 (n = 268).

c Moderately low functional exercise capacity (second quartile): DASI 15.45 < 24.95 (n = 248).

d Low functional exercise capacity (first quartile): DASI 0 < 15.45 (n = 252).

### Prognostic accuracy

**Figure 4:**
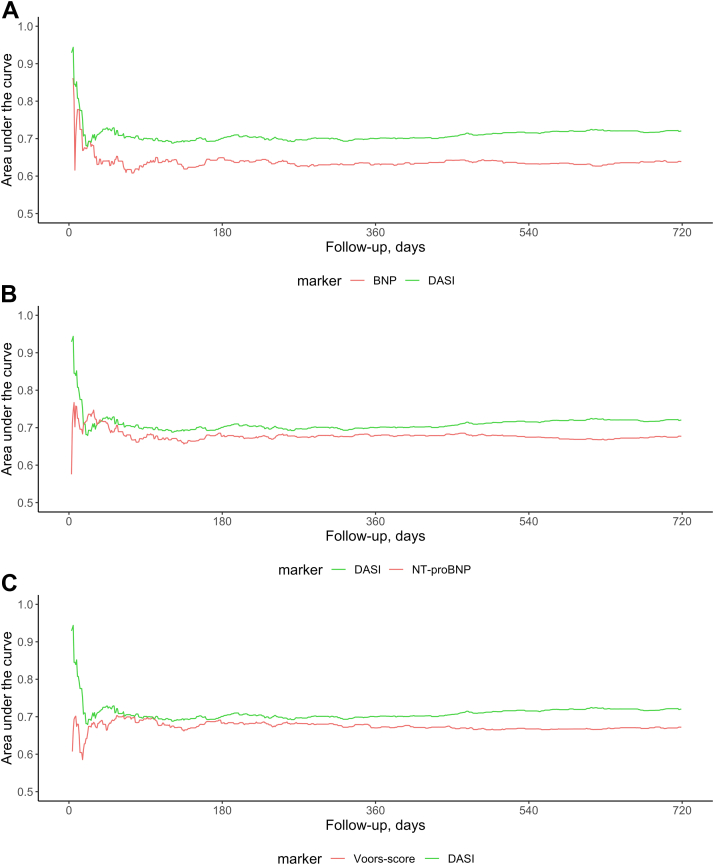
**Prognostic Accuracy of DASI for All-Cause Mortality Within 720 days of Follow-Up Compared to Natriuretic Peptide Peptide Concentrations on Admission and to the Voors Score** Time-dependent area under the receiver operating characteristics curve within 720 days of follow-up in patients presenting with acute dyspnea using the DASI, B-type natriuretic peptide (BNP) **(A)**, N-terminal pro-B-type natriuretic peptide (NT-proBNP) **(B)** levels on admission, and the Voors score **(C)** with previously described cut-off points (NT-proBNP >4,000 pg/mL, blood urea nitrogen (BUN) >11 mmol/L, age >70 years, hemoglobin <12 g/dL, and beta-blocker use at baseline). Calculated with “timeROC” package R statistical software. AUROC = area under the receiver operating characteristics curve; BNP = B-type natriuretic peptide; BUN = blood urea nitrogen; DASI = Duke Activity Status Index; NT-proBNP = N-terminal pro-B-type natriuretic peptide.

The prognostic accuracy of the DASI score was high, and higher compared to BNP and NT-proBNP levels on admission: for 720-day mortality prediction C-index 0.67 vs 0.62 (n = 743; *P* = 0.024) and 0.69 vs 0.65 (n = 998; *P* = 0.075). The prognostic performance of DASI was significantly higher than that of the previously described Voors score (C-index 0.68 vs 0.64, n = 854; *P* < 0.001). These findings were also reported using time-dependent area under the receiver operating characteristic curve (Figure 4) and Kaplan–Meier curves (Supplemental Figure 2). The fully adjusted model showed a high accuracy (C-index of 0.75). Calibration plots display observed vs predicted mortality probability (Supplemental Figures 3 and 4). Corrected C-indices and estimates for optimism are part of the supplement (Supplemental Table 5). The clinical usefulness was assessed by decision curve analysis. Supplemental Figure 5 compares the clinical net benefit for prediction of 720-day all-cause mortality of BNP concentration on admission, the Voors score, DASI quartiles, and DASI score to the extreme cases of intervention for none or all patients. This decision curve analysis showed that for a threshold probability of 0.3 the DASI score (net benefit 0.093) predicted 49 more true positives per 1,000 patients compared to BNP (net benefit 0.044).

### Subgroup Analysis

Similar findings to those of the overall cohort were observed in the 529 (52%) patients with an adjudicated final diagnosis of AHF, the aHR for 90- and 720-day mortality increased continuously from the fourth (reference) to the first DASI quartile (Supplemental Tables 6 and 7). Again, the prognostic accuracy of the DASI score for 90- and 720-day mortality was moderate (C-index 0.65 and 0.64) (Supplemental Figure 6). There was no significant interaction between DASI and gender (Supplemental Table 8).

## Discussion

This study directly compared the prognostic accuracy of quantified, self-reported exercise capacity using the DASI with the established objective disease-severity BNP in a large prospective multicenter study of patients presenting with acute dyspnea to the ED. We found that the patients in the lowest DASI quartile (0-15.25), and therefore with the lowest exercise capacity, were at significantly higher risk of death within 90 as well as 720 days, with a cumulative mortality of almost 50% after 720 days. Furthermore, the prognostic accuracy of the DASI score for all-cause mortality was high, and higher than BNP as a well-validated possible reference standard. It was even numerically higher vs NT-proBNP concentrations, a biomarker that also includes aspects of renal dysfunction as a second prognostic variable.^13^ The prognostic value of the DASI persisted after multivariable adjustment for other prognostic variables including age, sex, history of heart failure, chronic obstructive pulmonary disease, pulse oximetry, serum creatinine, hemoglobin, and NT-proBNP concentrations. Patients with low exercise capacity in the days preceding hospitalization (first DASI quartile) had a 7-fold higher risk of death within 720 days vs patients with high exercise capacity (fourth DASI quartile). Comparing the prognostic performance between DASI and an established mortality risk score for patients with heart failure (Voors score) showed a significantly higher performance, emphasizing the substantial prognostic value of self-reported exercise capacity as quantified by DASI. These findings were consistent in the subgroup with an adjudicated final diagnosis of AHF. Furthermore, no sex modifying interactions were found indicating a gender independent reliability.

These findings extend and corroborate previous insights obtained with the DASI in stable ambulatory patients, including patients scheduled for elective cardiac or major noncardiac surgery.^5^^,^^29^, ^30^, ^31^, ^32^, ^33^ In a large study including 1,401 patients undergoing major non-cardiac surgery, DASI scores even outperformed cardiopulmonary exercise testing and NT-proBNP concentrations in the prediction of death or myocardial infarction within 30 days of surgery.^5^^,^^10^ Given the facts that its prognostic value seemed to be largely independent of the acute disease causing the acute dyspnea (cardiac vs pulmonary) in the ED setting, sex had no modifying effect and it was also independent of the dominant comorbidity in the preoperative setting, the generalizability of implementing the DASI into clinical routine seems high. As the questions within the DASI substantially overlap with the initial nurse-lead functional and social assessment routinely performed in hospitalized patients in many institutions, the extra workload associated with the clinical implementation of the DASI may be rather small, further increasing the attractiveness of this tool. Prospective implementation studies seem warranted to evaluate the best strategies on how to implement the DASI into clinical routine.

In this study of mostly older patients presenting with acute dyspnea to the ED, the question related to sexual activity severely offended 2 patients leading to the withdrawal of this question for all remaining patients. As assessing sexual activities may be delicate and also controversial in many other settings and not all patients are sexual active with partners, the findings of this study, based on the modified DASI version without the question on sexual relations, may have major clinical implications as they highlight the opportunity of using this modified, so far not validated DASI version also elsewhere. Although, a recent study assessing whether all 12 DASI items were equally important, omitted the same sensitive question and showed that the discriminative ability of the DASI was not substantially weakened, further studies with the modified DASI version externally validating our findings are warranted.^34^

### Study Limitations

As this study required informed consent, we cannot comment on the possible value of the DASI in critically ill patients unable to provide informed consent. However, risk stratification is usually straight forward in critically ill patients. In addition, only about one-half of patients enrolled in BASEL V completed the DASI questionnaire shortly after presentation and were eligible for this analysis. Although their baseline characteristics were comparable to those who did not, we can only speculate on the generalizability of our findings to those patients. However, also in clinical practice, the possible clinical value of this instrument is restricted to those patients willing to complete DASI questionnaire, although the lack of participation might be a prognostic factor itself.^35^ This and whether an external estimation of answers to DASI questions by, eg, a caregiver, in particular in patients with long-term care, could be a reliable representation of a patient’s subjective exercise capacity, which is unknown and should be addressed in future studies. Finally, this was an observational study and therefore residual confounding should be taken into consideration.

## Conclusions

Quantification of self-reported exercise capacity with the use of DASI provided moderate to high prognostic accuracy for 90- and 720-day all-cause death in patients presenting with acute dyspnea to the ED, which was even higher than that of BNP as an established objective disease-severity marker. Obtaining the DASI may therefore aid physicians in patient-risk stratification.

PERSPECTIVES **COMPETENCY IN PATIENT CARE AND PROCEDURAL SKILLS:** Quantification of self-reported exercise capacity by the DASI provided high prognostic accuracy for short- and long-term mortality in patients with acute dyspnea presenting to the emergency department. The prognostic accuracy was even higher as compared to BNP concentrations.

**TRANSLATIONAL OUTLOOK:** The DASI may aid physicians in the risk stratification of patients presenting with acute dyspnea beyond providing important insights regarding functional health-related quality of life.

## Funding support and author disclosures

This study was supported by research grants from the 10.13039/501100001711Swiss National Science Foundation, the 10.13039/501100004362Swiss Heart Foundation, the 10.13039/100008375University of Basel, the 10.13039/100016015University Hospital Basel, Critical Diagnostics, 10.13039/100000046Abbott, BRAHMS, 10.13039/100004337Roche and 10.13039/100016946Singulex. None of those supporters had any role in study design, the conduct of the study, the analysis of the data, or the decision to submit this manuscript for publication. Dr Breidthardt has received research grants from the Swiss National Science Foundation, the University Hospital Basel, the Department of Internal Medicine, University Hospital Basel, Abbott, and Roche. Dr Mueller has received research grants from the Swiss National Science Foundation, the Swiss Heart Foundation, the KTI, the University Hospital Basel, the University of Basel, Abbott, Beckman Coulter, Biomerieux, BRAHMS, Ortho Clinical, Quidel, Novartis, Roche, Siemens, Singulex, and Sphingotec. The sponsors had no role in the design and conduct of the study; collection, management, analysis, and interpretation of the data; and preparation, review, or approval of the manuscript. All other authors have reported that they have no relationships relevant to the contents of this paper to disclose.
