# Supplementary material for: Prognostic Value of Self-Reported Subjective Exercise Capacity in Patients With Acute Dyspnea
Source: JACC Adv. 2023 May 26;2(3):100342. doi: 10.1016/j.jacadv.2023.100342 (PMC11198416; doi:10.1016/j.jacadv.2023.100342)
Supplement: Supplemental Figure 1-6 and Tables 1-8 [file mmc1.docx]

**Supplemental Table 1.** Comparison of patients with and without completed Duke Activity Status Index.

| **Supplemental Table 1. Comparison of patients with and without completed Duke Activity Status Index.** | | | | |
| --- | --- | --- | --- | --- |
|  | All patients n=2153 | Excluded n=1134 | Included n=1019 | p-value |
| Age, years | 75.0 [61.0, 82.0] | 75.0 [62.0, 82.0] | 74.0 [61.0, 82.0] | 0.196 |
| Female gender, no. (%) | 951 (44) | 515 (45) | 436 (43) | 0.237 |
| Body mass index, kg/m^2^ | 25.9 [22.5, 30.0] | 25.9 [22.5, 29.7] | 25.9 [22.3, 30.1] | 0.773 |
| History, no. (%) |  |  |  |  |
| Hypertension | 1451 (67) | 762 (67) | 689 (68) | 0.942 |
| Diabetes | 493 (23) | 253 (22) | 240 (24) | 0.526 |
| Coronary artery disease | 718 (33) | 363 (32) | 355 (35) | 0.193 |
| Atrial fibrillation | 580 (27) | 279 (25) | 301 (30) | 0.011 |
| COPD/asthma | 720 (33) | 360 (32) | 360 (35) | 0.087 |
| Vital signs and symptoms on admission |  |  |  |  |
| Systolic blood pressure, mmHg | 137 [121, 155] | 137 [120, 155] | 138 [122, 155] | 0.638 |
| Heart rate, beats per minute | 90 [75, 106] | 90 [75, 105] | 91 [76, 107] | 0.688 |
| SpO2, % | 96 [93, 98] | 96 [93, 98] | 96 [93, 98] | 0.330 |
| Body temperature, °C | 37.1 [36.7, 37.6] | 37.1 [36.6, 37.7] | 37.1 [36.7, 37.6] | 0.298 |
| Leg edema, no. (%) | 870 (41) | 440 (40) | 430 (42) | 0.228 |
| First in-hospital laboratory findings |  |  |  |  |
| BUN, mmol/L | 7.4 [5.2, 11.2] | 7.4 [5.2, 11.5] | 7.4 [5.3, 10.9] | 0.979 |
| Hemoglobin, g/L | 133 [118, 146] | 133 [120, 145] | 133 [117, 146] | 0.592 |
| Creatinine, µmol/L | 88 (69-119) | 89 (69-130) | 97 (73-135) | 0.002 |
| Sodium, mmol/L | 138 [136, 141] | 138 [135, 141] | 139 [136, 141] | 0.020 |
| Potassium, mmol/L | 4.1 [3.8, 4.4] | 4.0 [3.8, 4.4] | 4.1 [3.8, 4.4] | 0.152 |
| NT-proBNP, ng/L | 1273 [220, 5070] | 1308 [220, 5053] | 1221 [221, 5142] | 0.968 |
| Medication on admission, no. (%) |  |  |  |  |
| ACEI/ ARB | 1056 (49) | 540 (48) | 516 (51) | 0.228 |
| β-blocker | 919 (43) | 477 (43) | 442 (43) | 0.690 |
| Diuretics | 1093 (51) | 564 (50) | 529 (52) | 0.445 |

Comparison of characteristics at baseline of patients who were included in this analysis (n=1019) and patients who were excluded (n=1134). Inclusion criteria were a completed Duke Activity Status Index within 30 days after presentation to the emergency department with an adjudicated cause of dyspnea. Values are median (interquartile range) or numbers (%).  P-values displayed are calculated Chi2 test for categorical and Mann-Whitney-U for continuous variables. P-values <0.05 were considered statistically significant. All hypothesis testing was two-tailed. ACEI: angiotensin converting enzyme inhibitor, ARB: aldosterone receptor blocker, BUN: blood urea nitrogen, COPD: chronic obstructive pulmonary disease, DASI: Duke Activity Status Index, NT-proBNP: N-terminal pro-B-type natriuretic peptide, SpO2: peripheral oxygen saturation.

**Supplemental Table 2. Overview of the covariates and summary statistics used for multivariable Cox regression models.**

|  | Analysis  n = 1019 | missing |
| --- | --- | --- |
| Age, years | 74 [61, 82] | 0% (0) |
| Female Sex, | 436 (43) | 0% (0) |
| Signs and symptoms on entry |  |  |
| Systolic blood pressure, mmHg | 138 [122,155] | <1% (2) |
| Peripheral oxygen saturation, % | 96 [93,98] | <1% (4) |
| Leg edema | 430 (42) | 0% (0) |
| History of |  |  |
| Hypertension | 689 (68) | 0% (0) |
| Diabetes | 240 (24) | 0% (0) |
| Coronary artery disease | 355 (35) | 0% (0) |
| Atrial fibrillation | 301 (30) | 0% (0) |
| Heart failure | 356 (35) | 0% (0) |
| COPD/asthma | 360 (35) | 0% (0) |
| First in-hospital laboratory findings |  |  |
| BUN, mmol/L | 7.4 [5.2, 11.1] | 12% (127) |
| Creatinine level, µmol/L | 89 [69, 119] | 1% (13) |
| Hemoglobin level, g/L | 133 [117, 146] | 2% (19) |
| NT-proBNP level, ng/L | 1221 [220, 5162] | 2% (21) |
| Sodium level, mmol/L | 139 [136, 141] | 2% (25) |
| Medication on entry |  |  |
| ACEI/ARB | 516 (51) | <1% (3) |
| β-blockers | 442 (43) | <1% (2) |
| Diuretics | 529 (52) | <1% (2) |

Missing values of patients included in this analysis (n=1019). Continuous variables are presented as medians with interquartile ranges, categorical variables as number (percent), number of missing as percent (count). COPD: chronic obstructive pulmonary disease, eGFR: estimated glomerular filtration rate as per CKD-EPI, NT-proBNP: N-terminal pro-B-type natriuretic peptide.

**Supplemental Table 3.** **Unadjusted and adjusted hazard ratios of Duke Activity Status Index for 90-day all-cause mortality.**

|  | **Univariable analyses** | | | **Multivariable analysis** | | | | |
| --- | --- | --- | --- | --- | --- | --- | --- | --- |
| Variables | HR | 95% CI | P-value | HR | 95% CI | P-value | |  |
| Age (years) | 1.034 | (1.015 - 1.054) | 0.001 | 1.012 | (0.991 - 1.034) | 0.266 |  |  |
| Female sex | 0.578 | (0.354 - 0.944) | 0.028 | 0.487 | (0.283 - 0.810) | 0.006 |  |  |
| Log-BUN (mmol/L) | 2.533 | (1.719 - 3.734) | <0.001 | 1.304 | (0.811 - 2.097) | 0.273 |  |  |
| Hemoglobin (g/L) | 0.974 | (0.964 - 0.983) | <0.001 | 0.984 | (0.973 - 0.994) | 0.003 |  |  |
| Log-NT-proBNP (ng/L) | 1.407 | (1.226 - 1.616) | <0.001 | 1.193 | (1.001 - 1.422) | 0.049 |  |  |
| Beta-blocker | 1.129 | (0.717 - 1.776) | 0.601 | 0.678 | (0.425 - 1.082) | 0.103 |  |  |
| **Reversed DASI-score ^*^** | **1.052** | **(1.032 - 1.071)** | **<0.001** | **1.044** | **(1.024 - 1.065)** | **<0.001** |  |  |

*higher score indicating lower exercise capacity

Univariable and multivariable Cox regression analyses for 90-day all-cause mortality with Duke Activity Status Index (DASI) treated as continuous variable (n=1019). Adjustments made for age (years), sex, natural log-transformed blood urea nitrogen (BUN, mmol/L), hemoglobin level (g/L), natural log-transformed N-terminal pro-B-Type natriuretic peptide (NT-proBNP, ng/L) concentrations at presentation and intake of beta-blockers on admission.

**Supplemental Table 4.** **Unadjusted and adjusted hazard ratios of Duke Activity Status Index for 720-**

|  | **Univariable analyses** | | | **Multivariable analysis** | | | |  |
| --- | --- | --- | --- | --- | --- | --- | --- | --- |
| Variables | HR | 95% CI | P-value | HR | 95% CI | | P-value | |
| Age (years) | 1.046 | (1.035 - 1.057) | <0.001 | 1.031 | (1.018 - 1.044) | <0.001 | |  |
| Female sex | 0.782 | (0.607 - 0.988) | 0.040 | 0.632 | (0.489 - 0.818) | <0.001 | |  |
| Systolic BP (mmHg) | 0.986 | (0.982 - 0.991) | <0.001 | 0.992 | (0.983 - 0.997) | 0.001 | |  |
| SpO2 (%) | 0.975 | (0.957 - 0.995) | 0.012 | 0.983 | (0.962 - 1.004) | 0.120 | |  |
| Leg edema | 1.428 | (1.138 - 1.793) | 0.002 | 0.786 | (0.615 - 1.004) | 0.054 | |  |
| Hypertension | 1.571 | (1.203 - 2.051) | 0.001 | 0.884 | (0.652 - 1.200) | 0.431 | |  |
| Diabetes | 1.289 | (1.001 - 1.661) | 0.049 | 0.965 | (0.726 - 1.284) | 0.809 | |  |
| Coronary artery disease | 1.748 | (1.391 - 2.196) | <0.001 | 1.045 | (0.796 - 1.373) | 0.751 | |  |
| Atrial fibrillation | 1.700 | (1.347 - 2.145) | <0.001 | 0.962 | (0.734 - 1.259) | 0.776 | |  |
| History of heart failure | 2.076 | (1.653 - 2.607) | <0.001 | 1.011 | (0.756 - 1.353) | 0.939 | |  |
| COPD/asthma | 1.169 | (0.926 - 1.476) | 0.189 | 1.097 | (0.855 - 1.408) | 0.465 | |  |
| Log-BUN (mmol/L) | 2.073 | (1.704 - 2.522) | <0.001 | 1.208 | (0.863 - 1.692) | 0.270 | |  |
| Creatinine (µmol/L) | 1.003 | (1.002 - 1.005) | <0.001 | 0.999 | (0.996 - 1.001) | 0.395 | |  |
| Hemoglobin (g/L) | 0.981 | (0.976 - 0.986) | <0.001 | 0.991 | (0.985 - 0.997) | 0.002 | |  |
| Log-NT-proBNP (ng/L) | 1.353 | (1.266 - 1.447) | <0.001 | 1.184 | (1.076 - 1.302) | 0.001 | |  |
| Sodium (mmol/l) | 0.983 | (0.957 - 1.009) | 0.188 | 0.984 | (0.958 - 1.011) | 0.244 | |  |
| ACE/ARB | 1.271 | (1.010 - 1.598) | 0.041 | 0.738 | (0.569 - 0.958) | 0.022 | |  |
| Beta-blocker | 1.540 | (1.226 - 1.935) | <0.001 | 1.006 | (0.775 - 1.306) | 0.961 | |  |
| Diuretics | 2.365 | (1.845 - 3.031) | <0.001 | 1.273 | (0.936 - 1.732) | 0.124 | |  |
| **Reversed DASI-score*** | **1.049** | **(1.039 - 1.058)** | **<0.001** | **1.040** | **(1.031 - 1.050)** | **<0.001** | |  |
| *higher score indicating lower exercise capacity  Univariable and multivariable Cox regression analyses for 720-day all-cause mortality with Duke Activity Status Index (DASI) treated as continuous variable (n=1019). Adjustments made for age (years), sex, systolic blood pressure (mmHg) and peripheral oxygen saturation (%) on presentation, leg edema, history of hypertension, diabetes mellitus, coronary artery disease, atrial fibrillation, heart failure and chronic obstructive lung disease (COPD)/asthma, natural log-transformed blood urea nitrogen (BUN, mmol/L), serume creatinine level (µmol/L), hemoglobin level (g/L natural log-transformed N-terminal pro-B-Type natriuretic peptide (NT-proBNP, ng/L) concentrations and sodium level (mmol/l) at presentation and intake of angiotensin converting enzyme inhibitors/angiotensin receptor blockers (ACEI/ARB), beta-blockers and diuretics at admission. | | | | | | | | |

**day all-cause mortality.**

**Supplemental Table 5.** **C-indices with** **estimation of optimism.**

| Model | Outcome | C-Index | Dxy  (org) | Dxy  (training) | Dxy  (test) | Dxy (optimism) | Dxy  (corrected) |
| --- | --- | --- | --- | --- | --- | --- | --- |
| DASI-score  (n=743) | 720d  mortality | 0.67 | 0.346 | 0.343 | 0.346 | -2.60E-3 | 0.348 |
| BNP  (n=743) | 720d  mortality | 0.62 | 0.236 | 0.236 | 0.236 | 8.64E-4 | 0.235 |
| DASI-score  (n=998) | 720d  mortality | 0.68 | 0.380 | 0.382 | 0.380 | 2.33E-3 | 0.377 |
| NT-proBNP (n=998) | 720d  mortality | 0.65 | 0.307 | 0.304 | 0.307 | -2.54E-3 | 0.310 |
| DASI-  score  (n=854) | 720d  mortality | 0.68 | 0.365 | 0.364 | 0.365 | -1.58E-3 | 0.367 |
| Voors-score  (n=854) | 720d  mortality | 0.64 | 0.293 | 0.298 | 0.290 | 4.48E-3 | 0.286 |
| Full adjusted Model  (single imputed dataset) | 720d  mortality | 0.75 | 0.536 | 0.549 | 0.521 | 28.4E-3 | 0.509 |

Dxy: Somers’ Dxy rank correlation

org: Dxy of model fitted on original data

training: Dxy of model fitted on bootstrap sample

test: Dxy of model, which was fitted on bootstrap sample, evaluated on original data

optimism: average difference between Dxy training and Dxy test

corrected: optimism corrected Dxy=Dxy_org –Dxy_optimism

BNP: B-type natriuretic peptide.

DASI: Duke Activity Status Index

NT-proBNP: N-terminal pro-B-type natriuretic peptide.

**Supplemental Table 6.** **Adjusted hazard ratios of Duke Activity Status Index in patients with acute heart failure for 90-day all-cause mortality.**

|  | **Multivariable analysis** | | | **Multivariable analysis** | | | | |
| --- | --- | --- | --- | --- | --- | --- | --- | --- |
| Variables | HR | 95% CI | P-value | HR | 95% CI | P-value | |  |
| Age (years) | 1.038 | (1.004 - 1.073) | 0.027 | 1.036 | (1.003 - 1.071) | 0.034 |  |  |
| Female sex | 0.539 | (0.284 - 1.025) | 0.059 | 0.535 | (0.282 - 1.015) | 0.056 |  |  |
| Log-BUN (mmol/L) | 1.250 | (0.682 - 2.294) | 0.470 | 1.259 | (0.688 - 2.304) | 0.456 |  |  |
| Hemoglobin (g/L) | 0.989 | (0.975 - 1.004) | 0.149 | 0.217 | (0.977 - 1.005) | 0.001 |  |  |
| Log-NT-proBNP (ng/L) | 1.275 | (0.952 - 1.707) | <0.001 | 1.286 | (0.959 - 1.726) | 0.093 |  |  |
| Beta-blocker | 1.139 | (0.617 - 2.100) | 0.678 | 1.126 | (0.611 - 2.073) | 0.704 |  |  |
| **DASI 4th quartile*** | **reference** | |  |  |  |  |  |  |
| **DASI 3rd quartile** ^†^ | **2.032** | **(0.690 - 5.982)** | **0.198** |  |  |  |  |  |
| **DASI 2nd quartile** ^‡^ | **2.045** | **(0.701- 5.963)** | **0.190** |  |  |  |  |  |
| **DASI 1st quartile** ^§^ | **3.545** | **(1.305- 9.629)** | **<0.001** |  |  |  |  |  |
| **Reversed DASI-score**^ll^ |  |  |  | **1.036** | **(1.011 - 1.063)** | **0.005** |  |  |

*high functional exercise capacity (4^th^ quartile): DASI 32.45-52.95 (n=138)

^†^moderately high functional exercise capacity (3^rd^ quartile): DASI 21.45<32.45 (n=126)

^‡^moderately low functional exercise capacity (2^nd^ quartile): DASI 13.7<21.45(n=132)

^§^low functional exercise capacity (1^st^ quartile): DASI 0<13.7 (n=133)

^ll^ higher DASI-score indicating lower exercise capacity

Multivariable Cox regression analyses for 90-day all-cause mortality with Duke Activity Status Index (DASI) stratified into quartiles and treated as continuous variable (n=529). DASI quartiles referenced to 4^th^ quartile. Adjustments made for age (years), sex, natural log-transformed blodd urea nitrogen (BUN, mmol/L), hemoglobin level (g/L), natural log of N-terminal pro-B-Type natriuretic peptide (NT-proBNP, ng/L) concentrations at presentation and intake of beta-blockers on admission.**Supplemental Table 7.** **Adjusted hazard ratios of Duke Activity Status Index in patients with acute**

|  | | **Multivariable analysis** | | | **Multivariable analysis** | | | |  |
| --- | --- | --- | --- | --- | --- | --- | --- | --- | --- |
| Variables | HR | | 95% CI | P-value | HR | 95% CI | | P-value | |
| Age (years) | 1.049 | | (1.031 - 1.068) | <0.001 | 1.049 | (1.031 - 1.068) | <0.001 | |  |
| Female sex | 0.745 | | (0.534 - 1.039) | 0.083 | 0.713 | (0.511 - 0.994) | <0.001 | |  |
| Systolic BP (mmHg) | 0.987 | | (0.981 - 0.994) | <0.001 | 0.978 | (0.954 - 1.004) | 0.095 | |  |
| SpO2 (%) | 0.976 | | (0.951 - 1.002) | 0.066 | 0.816 | (0.599 - 1.112) | 0.198 | |  |
| Leg edema | 0.820 | | (0.599 - 1.123) | 0.215 | 0.894 | (0.593 - 1.348) | 0.593 | |  |
| Hypertension | 0.915 | | (0.607 - 1.378) | 0.669 | 0.894 | (0.593 - 1.348) | 0.593 | |  |
| Diabetes | 1.084 | | (0.770 - 1.526) | 0.643 | 1.091 | (0.776 - 1.534) | 0.617 | |  |
| Coronary artery disease | 1.098 | | (0.782- 1.542) | 0.589 | 1.078 | (0.770 - 1.509) | 0.661 | |  |
| Atrial fibrillation | 0.976 | | (0.708 - 1.345) | 0.883 | 0.938 | (0.683 - 1.287) | 0.690 | |  |
| History of heart failure | 1.115 | | (0.778- 1.598) | 0.552 | 1.097 | (0.765 - 1.575) | 0.614 | |  |
| COPD/asthma | 1.346 | | (0.978 - 1.852) | 0.068 | 1.321 | (0.960 - 1.819) | 0.088 | |  |
| Log-BUN (mmol/L) | 1.320 | | (0.865 - 2.013) | 0.198 | 1.276 | (0.842 - 1.934) | 0.251 | |  |
| Creatinine (µmol/L) | 1.000 | | (0.997 - 1.002) | 0.793 | 1.000 | (0.997 - 1.003) | 0.903 | |  |
| Hemoglobin (g/L) | 0.996 | | (0.988 - 1.003 | 0.257 | 0.996 | (0.989 - 1.004) | 0.347 | |  |
| Log-NT-proBNP (ng/L) | 1.247 | | (1.266 - 1.447) | <0.001 | 1.255 | (1.073 - 1.467) | 0.004 | |  |
| Sodium (mmol/l) | 0.983 | | (1.066- 1.460 | 0.006 | 0.998 | (0.964 - 1.033) | 0.904 | |  |
| ACE/ARB | 0.773 | | (0.556 - 1.076) | 0.127 | 0.788 | (0.567 - 1.094) | 0.154 | |  |
| Beta-blocker | 1.118 | | (0.809 - 1.547) | 0.499 | 1.147 | (0.831 - 1.584) | 0.404 | |  |
| Diuretics | 2.262 | | (0.819 - 1.943) | 0.291 | 1.276 | (0.829 - 1.966) | 0.268 | |  |
| **DASI 4th quartile*** | **reference** | |  |  |  |  |  | |  |
| **DASI 3rd quartile** ^†^ | **1.651** | | **(0.972 - 2.805)** | **0.064** |  |  |  | |  |
| **DASI 2nd quartile** ^‡^ | **2.596** | | **(1.593 - 4.232)** | **<0.001** |  |  |  | |  |
| **DASI 1st quartile** ^§^ | **2.930** | | **(1.811 - 4.740)** | **<0.001** |  |  |  | |  |
| **Reversed DASI-score**^ll^ |  | |  |  | **1.035** | **(1.022 - 1.048)** | **<0.001** | |  |
| *high functional exercise capacity (4^th^ quartile): DASI 32.45-52.95 (n=138)  ^†^moderately high functional exercise capacity (3^rd^ quartile): DASI 21.45<32.45 (n=126)  ^‡^moderately low functional exercise capacity (2^nd^ quartile): DASI 13.7<21.45(n=132)  ^§^low functional exercise capacity (1^st^ quartile): DASI 0<13.7 (n=133)  ^ll^higher DASI-score indicating lower exercise capacity  Multivariable Cox regression analyses for 720-day all-cause mortality with Duke Activity Status Index (DASI) stratified into quartiles and treated as continuous variable (n=529). Adjustments made for age (years), sex, systolic blood pressure (mmHg) and peripheral oxygen saturation (%) on presentation, leg edema, history of hypertsion, diabetes mellitus, coronary artery disease, atrial fibrillation, heart failure and chronic obstructive lung disease (COPD)/asthma, natural log of blood urea nitrogen (BUN, mmol/L), creatinine level (µmol/L), hemoglobin level (g/L), natural log of N-terminal pro-B-Type natriuretic peptide (NT-proBNP, ng/L) concentrations and sodium level (mmol/l) at presentation and intake of angiotensin converting enzyme inhibitors/angiotensin receptor blockers (ACEI/ARB), beta-blockers and diuretics at admission. | | | | | | | | | |

**heart failure for 720-day all-cause mortality.**

**Supplemental Table 8.** **Gender interaction analyses of Duke Activity Status Index for A 720-day all-cause mortality, B 90-day all-cause mortality and C 90-day mortality in patients with acute heart failure.**

**A** Variables P-value HR 95%CI

| Age (years) | .000 | 1.039 | 1.024 | 1.053 |
| --- | --- | --- | --- | --- |
| Female sex | .731 | .836 | .302 | 2.319 |
| Systolic BP (mmHg) | .014 | .993 | .988 | .999 |
| SpO2 (%) | .206 | .985 | .963 | 1.008 |
| Leg edema | .008 | .697 | .535 | .909 |
| Hypertension | .544 | .901 | .644 | 1.261 |
| Diabetes | .838 | .969 | .712 | 1.317 |
| Coronary artery disease | .777 | 1.043 | .778 | 1.400 |
| Atrial fibrillation | .516 | .909 | .681 | 1.213 |
| COPD/asthma | .209 | 1.187 | .908 | 1.551 |
| History of heart failure | .950 | .990 | .723 | 1.356 |
| Log-BUN (mmol/L) | .115 | 1.333 | .932 | 1.907 |
| Creatinine (µmol/L) | .318 | .999 | .996 | 1.001 |
| Hemoglobin (g/L) | .115 | .995 | .988 | 1.001 |
| Log-NT-proBNP (ng/L) | .000 | 1.210 | 1.087 | 1.346 |
| Sodium (mmol/l) | .211 | .982 | .954 | 1.010 |
| ACEI/ARB | .030 | .734 | .555 | .971 |
| Beta-blocker | .632 | 1.071 | .809 | 1.418 |
| Diuretics | .085 | 1.348 | .959 | 1.893 |
| DASI 4th quartile* | .000 |  |  |  |
| DASI 1^st^ quartile^§^ | .000 | 7.146 | 3.617 | 14.118 |
| DASI 2nd quartile^‡^ | .000 | 4.205 | 2.113 | 8.370 |
| DASI 3^rd^ quartile ^†^ | .011 | 2.505 | 1.235 | 5.083 |
| **Sex*DASI 4th quartile*** | .740 |  |  |  |
| **Sex*DASI 1^st^ quartile**^§^ | .480 | .673 | .224 | 2.022 |
| **Sex*DASI 2nd quartile**^‡^ | .833 | .887 | .291 | 2.707 |
| **Sex*DASI 3^rd^ quartile**^†^ | .487 | .656 | .199 | 2.158 |

**B** Variables P-value HR 95%CI

| Age (years) | .025 | 1.029 | 1.004 | 1.055 |
| --- | --- | --- | --- | --- |
| Female sex | .312 | .330 | .038 | 2.838 |
| Log-BUN (mmol/L) | .035 | 1.694 | 1.037 | 2.769 |
| Hemoglobin (g/L) | .097 | .990 | .978 | 1.002 |
| Log-NT-proBNP (ng/L) | .111 | 1.169 | .965 | 1.416 |
| Beta-blocker | .357 | .789 | .476 | 1.306 |
| DASI 4th quartile* | .003 |  |  |  |
| DASI 1st quartile^§^ | .018 | 3.295 | 1.225 | 8.866 |
| DASI 2nd quartile^‡^ | .531 | 1.408 | .483 | 4.104 |
| DASI 3rd quartile^†^ | .803 | .863 | .271 | 2.746 |
| **Sex*DASI 4th quartile*** | .878 |  |  |  |
| **Sex*DASI 1st quartile**^§^ | .608 | 1.807 | .188 | 17.363 |
| **Sex*DASI 2nd quartile**^‡^ | .958 | 1.070 | .088 | 13.024 |
| **Sex*DASI 3rd quartile**^†^ | .806 | 1.395 | .097 | 19.998 |

**C** Variables P-value HR 95%CI

| Age (years) | .030 | 1.038 | 1.004 | 1.074 |
| --- | --- | --- | --- | --- |
| Female sex | .714 | .653 | .067 | 6.377 |
| Log-BUN (mmol/L) | .183 | 1.522 | .820 | 2.824 |
| Hemoglobin (g/L) | .753 | .998 | .982 | 1.013 |
| Log-NT-proBNP (ng/L) | .055 | 1.356 | .994 | 1.850 |
| Beta-blocker | .637 | 1.169 | .610 | 2.239 |
| DASI 4th quartile* | .046 |  |  |  |
| DASI 1st quartile^§^ | .086 | 3.086 | .853 | 11.167 |
| DASI 2nd quartile^‡^ | .812 | 1.186 | .291 | 4.841 |
| DASI 3rd quartile^†^ | .874 | .890 | .211 | 3.757 |
| **Sex*DASI 4th quartile*** | .999 |  |  |  |
| **Sex*DASI 1st quartile**^§^ | .953 | .930 | .081 | 10.668 |
| **Sex*DASI 2nd quartile**^‡^ | .999 | .998 | .070 | 14.268 |
| **Sex*DASI 3rd quartile**^†^ | .970 | 1.055 | .064 | 17.312 |

**Supplemental Figure 1.** Patients flow diagram.


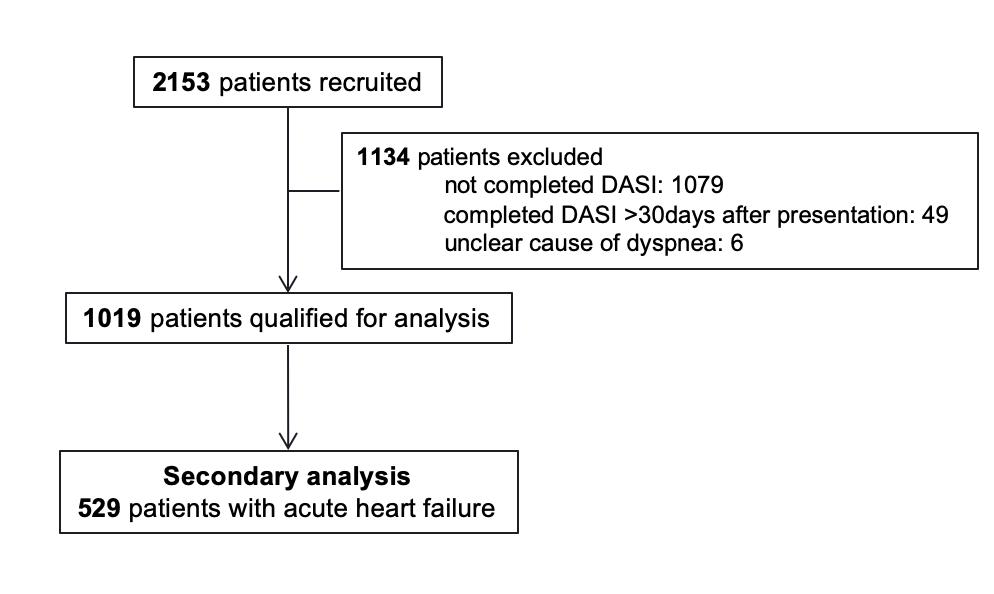


**Supplemental Figure 2. Kaplan-Meier survival curves for 720-day all-cause mortality in patients stratified to the Voors-score.**

**A**

**B**

**
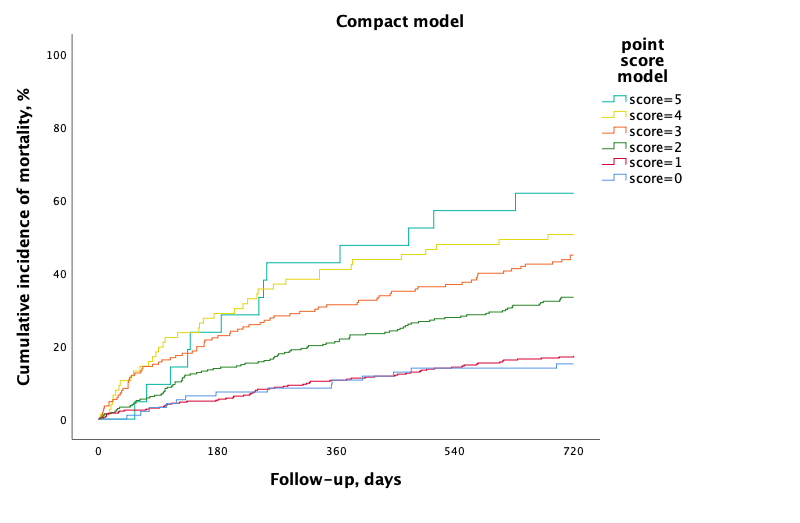
**

Kaplan-Meier survival curves for each score according to the Voors-score with previously described cut-off points for optimal classification derived from the compact model (NT-proBNP *>*4000 pg/mL, BUN *>*11 mmol/L, age *>*70 years, hemoglobin *<*12 g/dL and beta-blocker use at baseline. The scores subsequently ranges from 0 – 5. BUN: blood urea nitrogen, DASI: Duke Activity Status Index, NT-proBNP: N-terminal pro-B-type natriuretic peptide.

**Supplemental Figure 3. Calibration curves for 720-day all-cause mortality prediction of A BNP, B the Voors-score, B DASI-quartiles and D DASI-Score.**


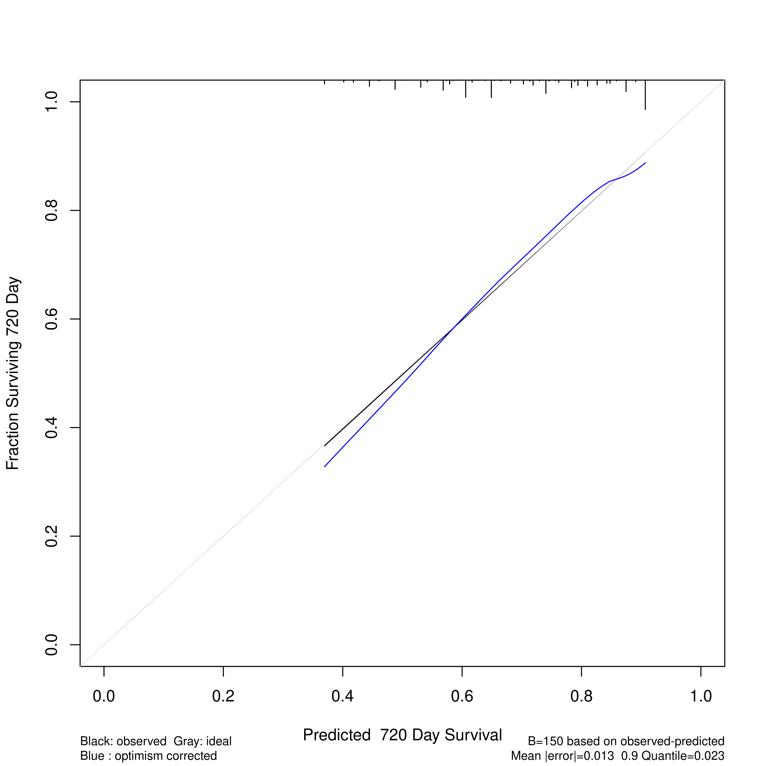

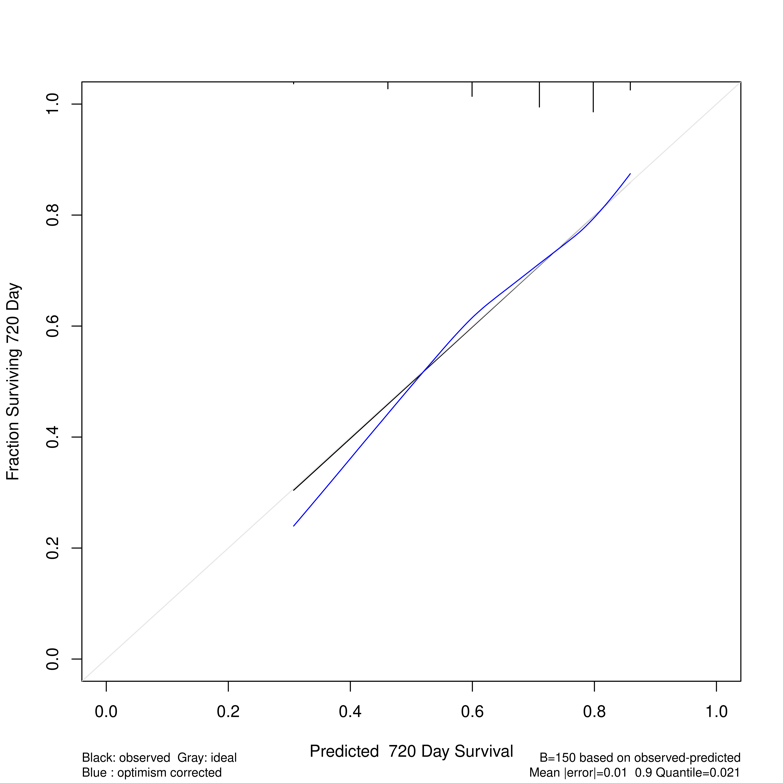

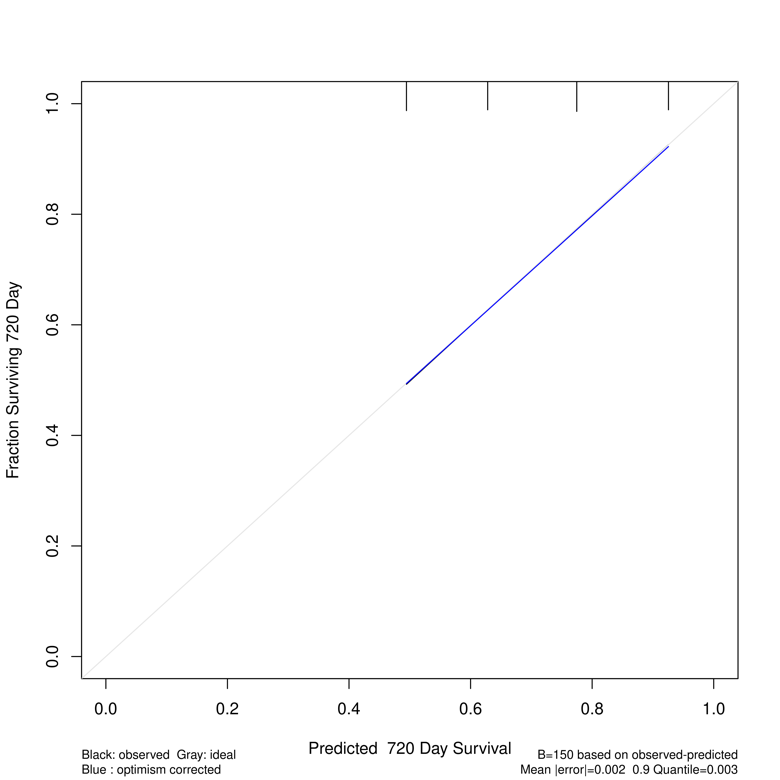

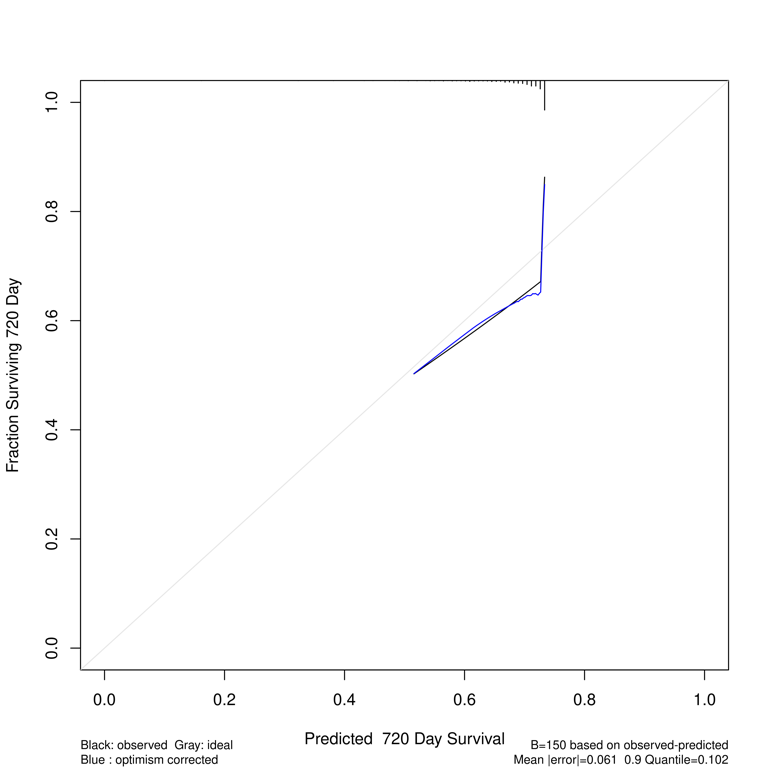


A

B

C

D

Calibration curves show observed versus predicted probability of 720-day all-cause mortality of A BNP (ng/L) concentration on admission, B the Voors-score, C DASI-quartiles and D DASI-Score. The gray linear line represents the ideal case where observed and predicted probability match. Black and blue curves display apparent and optimism corrected (bias corrected) performance, respectively. Calculated with “rms” package R statistical software. DASI: Duke Activity Status Index, BNP: B-type natriuretic peptide.

**Supplemental Figure 4. Calibration curve for 720-day all-cause mortality prediction of fully adjusted model.**

**
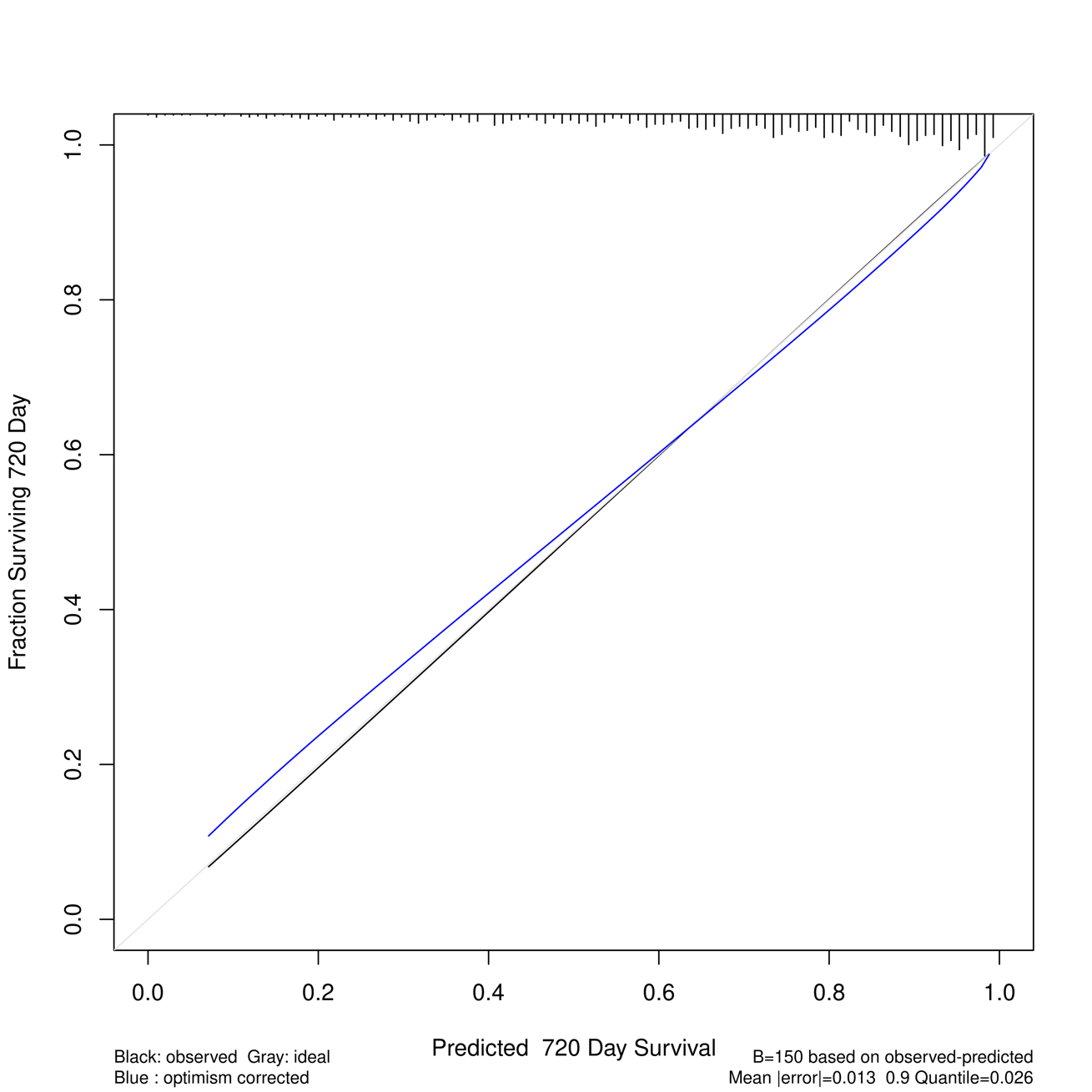
**

This calibration curve compares observed with predicted probability of 720-day all-cause mortality of the fully adjusted model. Variables used in this model are: sex, age, the natural logarithm blood urea nitrogen (BUN), hemoglobin level, the natural logarithm of N-terminal pro-B-type natriuretic peptide (NT-proBNP) concentration at presentation, beta-blocker intake on entry, systolic blood pressure and peripheral oxygen saturation at presentation, leg edema on entry, history of hypertension, diabetes mellitus, coronary artery disease, atrial fibrillation, previous heart failure and obstructive pulmonary disease, serum creatinine and sodium level, intake of angiotensin converting enzyme inhibitors/angiotensin receptor blockers, diuretics, and DASI-score. The gray linear line represents the ideal case where observed and predicted probability match. Black and blue curves display apparent and optimism corrected (bias corrected) performance, respectively. Calculated with “rms” package R statistical software. DASI: Duke Activity Status Index, NT-proBNP: NT-proBNP: N-terminal pro-B-type natriuretic peptide.

**
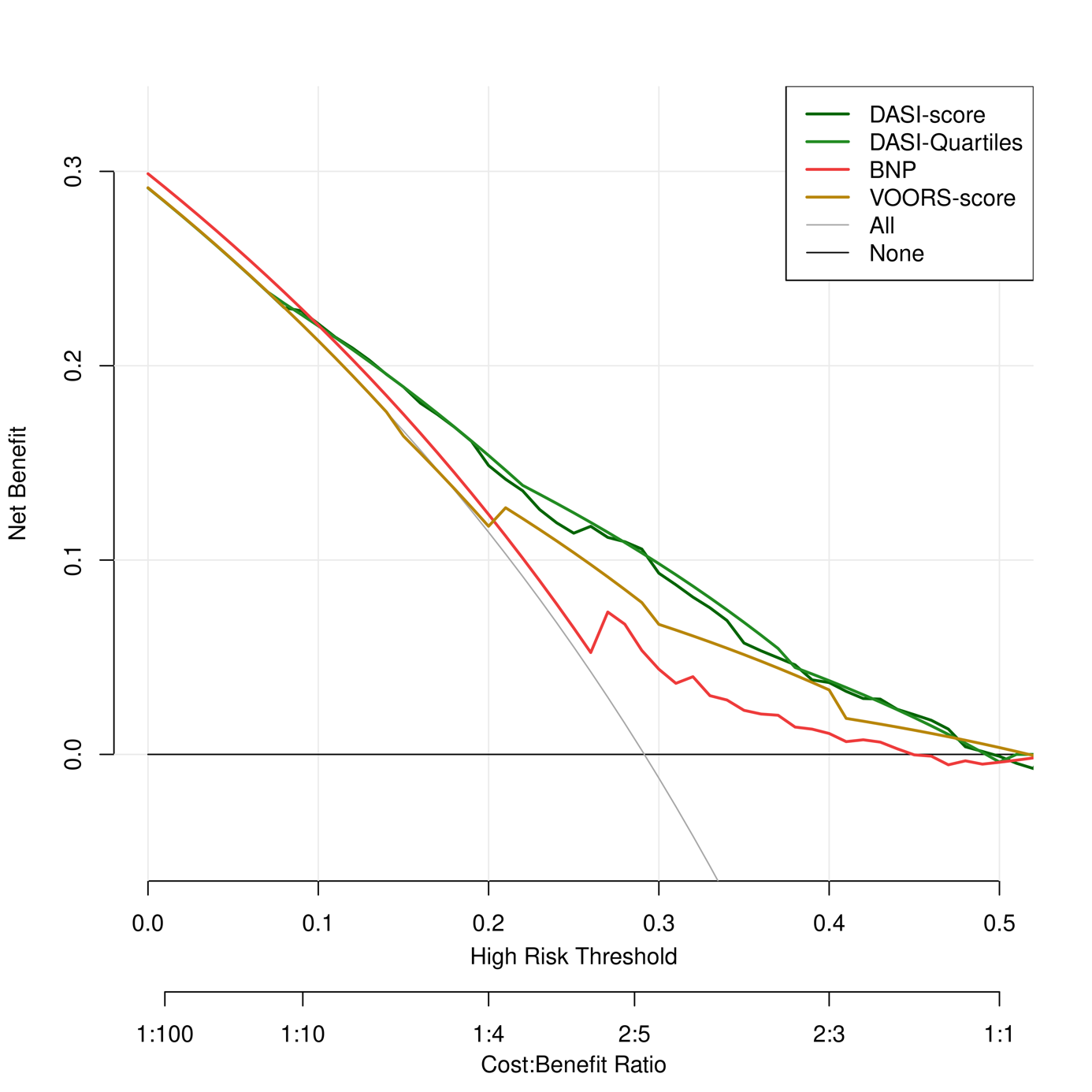
Supplemental Figure 5. Decision curve analysis for 720-day all-cause mortality prediction of A BNP, B the Voors-score, B DASI-quartiles and D DASI-Score.**

This decision curve analysis calculates the clinical net benefit for prediction of 720-day all-cause mortality of BNP (ng/L) concentration on admission (red line), the Voors-score (dark yellow line), DASI-quartiles (light green line) and DASI-score (dark green line). The curves are compared to the extreme cases of intervention for all patients (light grey transverse line) or intervention for none (black horizontal line). Net benefit: sum of true-positive minus false-positive classifications weighted by the respective threshold. For a specific threshold probability, a larger net benefit indicates a greater number of true positive predictions without increase of false positives. For a threshold probability of 0.3 which is equal to 30% or an odd of 3:7, one is willing to keep seven false-positive patients under observation to capture three true-positives. In this figure DASI-score (net benefit 0.093) predicts 49 more true positives per 1000 patients without increase in the rate of false positives compared to BNP (net benefit 0.044). The curves were obtained using the R package “rmda”. DASI: Duke Activity Status Index, BNP: B-type natriuretic peptide.

**Supplemental Figure 6**. **Prognostic accuracy of Duke Activity Status Index compared to BNP concentration on admission for all-cause mortality within 720 days of follow-up in patients with acute heart failure.**


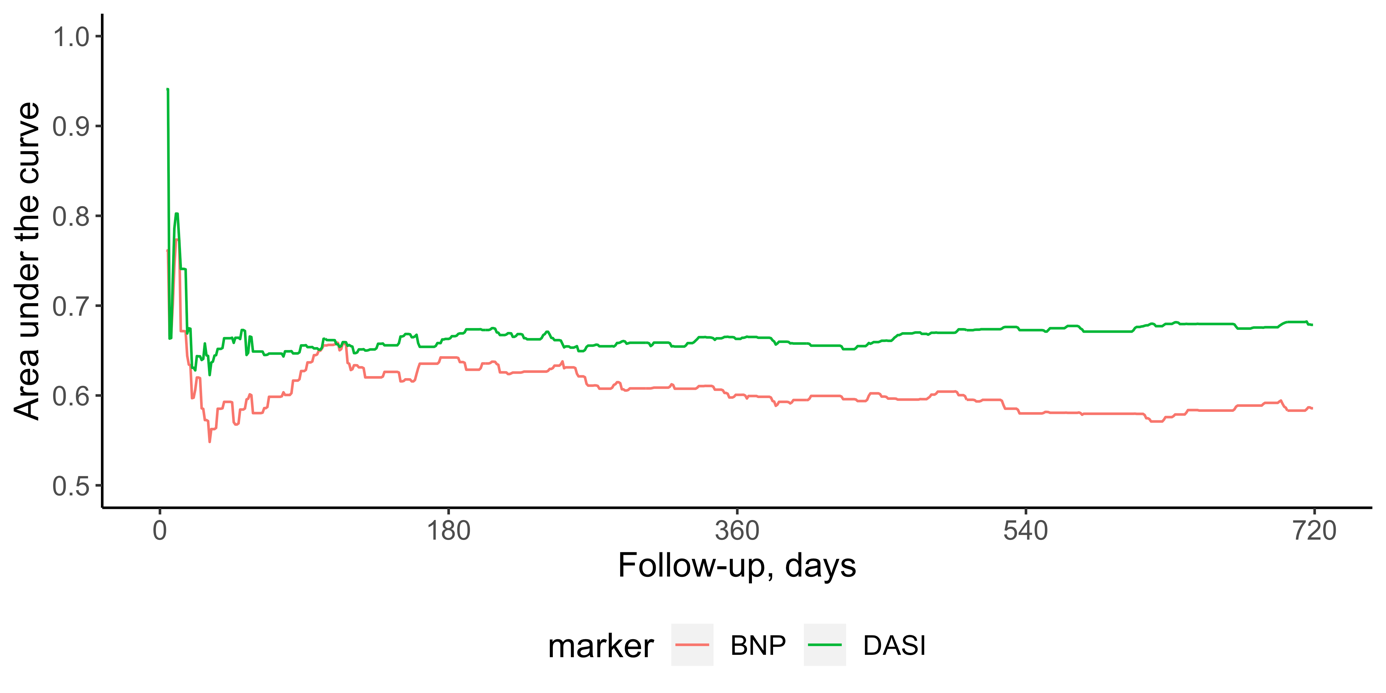


Time-dependent Area under the receiver Operating Characteristics curve (AUROC) within 720 days of follow-up in patients presenting with acute heart failure and available BNP (ng/L) concentration on admission. Calculated with “timeROC” package R statistical software. AUROC: Time-dependent Area under the receiver Operating Characteristics curve,DASI: Duke Activity Status Index, BNP: B-type natriuretic peptide.
